# P-1361. Cefiderocol Activity against *Pseudomonas aeruginosa*, Including Resistant Subsets and Isolates Carrying Carbapenemase β-lactamase Genes, from United States Hospitals (2020–2023)

**DOI:** 10.1093/ofid/ofae631.1538

**Published:** 2025-01-29

**Authors:** Rodrigo E Mendes, Joshua Maher, Cory Hubler, Abigail Scullin, Maura Karr, Hank Kimbrough, Mariana Castanheira

**Affiliations:** JMI Laboratories, North Liberty, Iowa; Element Materials Technology/Jones Microbiology Institute, North Liberty, Iowa; Element Materials Technology/Jones Microbiology Institute, North Liberty, Iowa; Element Materials Technology/Jones Microbiology Institute, North Liberty, Iowa; Element Materials Technology/Jones Microbiology Institute, North Liberty, Iowa; Element Materials Technology/Jones Microbiology Institute, North Liberty, Iowa; JMI Laboratories, North Liberty, Iowa

## Abstract

**Background:**

*Pseudomonas aeruginosa* (PSA) possess various intrinsic treatment-limiting resistance mechanisms, leading to decreased antibiotic permeability, and also the ability to acquire resistance. Cefiderocol (FDC) is a siderophore cephalosporin that uses the iron transport systems of Gram-negative bacteria to optimize cell entry. The activity of FDC and comparator agents were evaluated against PSA causing infections in US hospitals, including resistant subsets, as part of the SENTRY Antimicrobial Surveillance Program.

**Figure:**
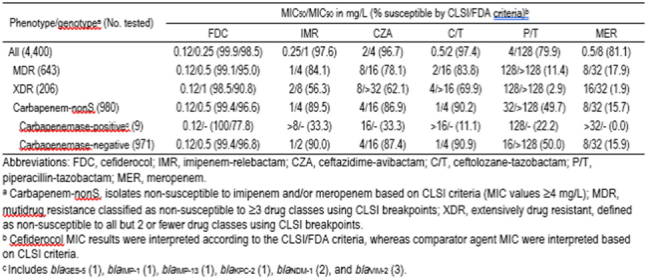

**Methods:**

4,400 PSA were collected from 36 sites in the US in 2020–2023. Susceptibility (S) testing was performed by broth microdilution with cation-adjusted Mueller-Hinton broth (CAMHB) for comparators and iron-depleted CAMHB for FDC. CLSI/FDA criteria were applied. Isolates with imipenem (IMI) or meropenem MIC ≥4 mg/L (nonS by CLSI) were screened for β-lactamase genes. Multidrug resistance (MDR) was defined as nonS to ≥3 drug classes by CLSI; whereas, extensively drug resistance (XDR) was defined as nonS to all but 2 or fewer drug classes.

**Results:**

14.6% (643/4,400) and 4.7% (206/4,400) PSA isolates were MDR and XDR, respectively. 22.3% (980/4,400) PSA were carbapenem-nonS, with only 9 (0.9%) strains carrying carbapenemases. FDC and β-lactam/β-lactamase inhibitor (BL/BLI) combinations showed S of > 95% against all PSA, except for piperacilin-tazobactam (79.9%S) (Table). FDC (90.8–99.4%) showed MIC_50_ of 0.12 mg/L and MIC_90_ of 0.5–1 mg/L against MDR, XDR and carbapenem-nonS isolates, whereas BL/BLI showed S of 2.9–90.2%. FDC (100%S) inhibited all PSA carrying carbapenemases at the CLSI S breakpoint of ≤2 mg/L. Other comparators showed S of < 35% against the latter subset. FDC (MIC_50/90_, 0.12/0.5 mg/L; 96.8–99.4%S) had MIC 4- to 8-fold lower than IMI-relebactam (MIC_50/90_, 1/2 mg/L; 90.0%S) and cetolozane-tazobactam (MIC_50/90_, 1/4 mg/L; 90.9%S), which were also active against carbapenem-nonS PSA absent of carbapenemase genes.

**Conclusion:**

FDC showed potent and cosistent activity against PSA clinical isolates from US hospitals, including resistant subsets and those with or without carbapenemase genes. These data demonstrate FDC activity against PSA resistant subsets, for which antibiotic treatment options are limited.

**Disclosures:**

**Rodrigo E. Mendes, PhD**, GSK: Grant/Research Support

